# EFFECTS OF CALCIUM SUPPLEMENTATION ON THE RISK OF FRACTURE IN OLDER ADULTS

**DOI:** 10.1590/1413-785220253306e284103

**Published:** 2025-11-10

**Authors:** Jianlei Li

**Affiliations:** 1 Shaoxing People's Hospital, Department of Orthopedics, Shaoxing, China.

**Keywords:** Calcium, Fracture, Older Adults, Meta-Analysis, Cálcio, Fratura, Idoso Fragilizado, Metanálise

## Abstract

To explore the effect of calcium supplementation on the risk of fractures at various sites in older adults based on randomized controlled trials (RCTs). PubMed, Embase, and the Cochrane Library were systematically searched for eligible RCTs from their inception until May 2023. The included trials investigated the effect of calcium supplementation on the risk of fracture in individuals aged 50 years or above, regardless of the use or nonuse of vitamin D. The primary and secondary outcome measures were total, vertebral, nonvertebral, and hip fractures. Twenty-three RCTs involving 70,837 individuals were enrolled. Calcium supplementation demonstrated a significant reduction in the risk of total (RR: 0.93; 95% CI: 0.88–0.99; P=0.019) and nonvertebral (RR: 0.93; 95% CI: 0.87–0.99; P=0.023) fractures. No significant differences were observed in vertebral (RR: 0.87; 95% CI: 0.75–1.01; P=0.074) and hip (RR: 0.90; 95% CI: 0.73–1.12; P=0.355) fractures between calcium and placebo or no treatment. Calcium dose influenced total fracture risk (P=0.008), while history of fracture (P=0.044), calcium dose (P=0.041), and follow-up duration (P=0.031) affected nonvertebral fracture risk. Follow-up duration impacted hip fracture risk (P=0.001). Calcium supplementation can significantly affect the risk of fracture, particularly nonvertebral fractures, in older adults. ***Level of Evidence I; Therapeutic Studies - Investigating the Results of Treatment*.**

## RESUMO

Explorar o efeito da suplementação de cálcio no risco de fraturas em vários locais em adultos mais velhos com base em ensaios clínicos randomizados (ECRs). A PubMed, Embase e a Biblioteca Cochrane foram sistematicamente pesquisadas para ensaios clínicos randomizados (ECRs) elegíveis desde a sua criação até maio de 2023. Os ensaios inclusos investigaram o efeito da suplementação de cálcio no risco de fraturas em indivíduos com 50 anos ou mais, independentemente do uso ou não de vitamina D. As medidas de desfecho primárias e secundárias foram fraturas totais, vertebrais, não vertebrais e do quadril. Vinte e três ECRs envolvendo 70.837 indivíduos foram incluídos. A suplementação de cálcio demonstrou uma redução significativa no risco de fraturas totais (RR: 0,93; IC 95%: 0,88–0,99; P=0,019) e não vertebrais (RR: 0,93; IC 95%: 0,87–0,99; P=0,023). Não foram observadas diferenças significativas nas fraturas vertebrais (RR: 0,87; IC 95%: 0,75–1,01; P=0,074) e do quadril (RR: 0,90; IC 95%: 0,73–1,12; P=0,355) entre cálcio e placebo ou nenhum tratamento. A dose de cálcio influenciou o risco de fratura total (P=0,008), enquanto a história de fratura (P=0,044), dose de cálcio (P=0,041) e duração do acompanhamento (P=0,031) afetaram o risco de fratura não vertebral. A duração do acompanhamento impactou o risco de fratura do quadril (P=0,001). A suplementação de cálcio pode afetar significativamente o risco de fratura, particularmente as fraturas não vertebrais, em adultos mais velhos. ***Nivel de Evidência l; Estudos terapêuticos - Investigação dos resultados do tratamento*.**

## INTRODUCTION

The prevalence of osteoporotic fracture has rapidly increased with the growth of population worldwide.^1^ The number of patients with osteoporosis in the European Union was estimated to be 27.5 million, whereas that of patients aged over 50 years in the USA reached 10.2 million.^2,3^ In the Asia-Pacific region, 5%–10% of adults have osteoporosis, with women aged ≥50 years exhibiting a higher prevalence.^4^ In Western countries, the lifetime risks of osteoporotic fracture in both men and women were 13%–22% and 40%–50%, respectively.^5^ Older adults with fractures experience significant harmful effects on their daily routines; even disability or mortality occurs in serious cases.^6,7^ Furthermore, fracture treatment is costly with the demographic trend of aging and the predicted increase in life expectancy.^8^

At present, calcium and vitamin D supplementation is widely recommended to reduce the risk of osteoporosis and progression of fractures. However, calcium supplementation may affect the risk of fracture; there is also a growing interest in the assessment of the role of vitamin D in bone health.^9^ In this study, we conducted a meta-analysis of randomized controlled trials (RCTs) to examine the effect of calcium supplementation on the risk of fracture and explored the potential role of the interaction between calcium and vitamin D in reducing the risk of fractures, including total, vertebral, nonvertebral, and hip fractures.

## MATERIALS AND METHODS

### Data sources, search strategy, and selection criteria

The Preferred Reporting Items for Systematic Reviews and Meta-Analyses was used in this study.^10^ Trials that compared the effects of calcium with those of placebo or no treatment on the risk of fractures in older adults were considered eligible, and no restriction on the publication status and languages was imposed. We systematically searched PubMed, Embase, and the Cochrane Library for eligible trials from their inception until May 2023, and the following terms were used: "calcium" [MeSH Terms] OR "calcium" [All Fields] AND ("fractures, bone" [MeSH Terms] OR ("fractures" [All Fields] AND "bone" [All Fields]) OR "bone fractures" [All Fields] OR "fracture" [All Fields]). Ongoing trials or additional eligible trials were also included if they met the abstract requirements, reference lists of relevant articles, and the clinicaltrials.gov website.

To avoid confounding biases, only RCTs were included. The inclusion criteria were as follows: (1) study design: RCT; (2) participants: individuals aged 50 years or above; (3) intervention: calcium supplementation, irrespective of vitamin D use; (4) control: placebo or no treatment; (5) follow-up: follow-up duration of more than 6 months; and (6) outcomes: total, vertebral, nonvertebral, and hip fractures.

### Data collection and quality assessment

The author independently extracted the following data: first author's name, publication year, country, sample size, age, male sex (%), history of fracture, vitamin D use, baseline 25 OHD, intervention, control, follow-up, and reported outcomes. Then, the quality of each trial was assessed using the Jadad scale based on the randomization used, data blinding, allocation concealment, withdrawals and dropouts of subjects, and use of intention-to-treat analysis.^11^ For each trial, the scoring system ranged from 0 to 5, and trials that scored 4 or 5 were considered to be of high quality. Any disagreement between the reviewers regarding data collection and quality assessment was settled by consulting an additional reviewer.

### Statistical analysis

The effects of calcium supplementation on the risk of fracture were used as categorical data, and the relative risk (RR) with 95% confidence interval (CI) was calculated before data pooling based on the events that occurred and the sample size of the intervention and control groups. The pooled RRs and 95% CIs for fracture risk were evaluated using the random-effects model, which considers the underlying variability across the included trials and provides conservative results.^12, 13^ Furthermore, heterogeneity across the included trials for each outcome was evaluated using the *I* ^2^ and Q statistics, and significant heterogeneity was defined as *I* ^2^ > 50.0% or *P* < 0.10.^14, 15^ Sensitivity analyses were conducted to assess the robustness of the pooled conclusions by sequentially removing individual trials.^16^ Subgroup analyses were also conducted based on the mean age, sex, history of fracture, baseline 25 OHD, calcium dose, vitamin D supplementation, follow-up duration, and study quality, and differences between the groups were evaluated using an interaction test, which was based on a *t-*test, and assuming a normal data distribution.^17^ Publication bias for each outcome was evaluated using qualitative (funnel plot) and quantitative (Egger's and Begg's tests) methods.^18,19^ The inspection level for the pooled outcome was two-sided, and *P* < 0.05 was considered to indicate statistical significance. All statistical analyses were conducted using the STATA software (version 10.0, Stata Corporation, College Station, TX, USA).

## RESULTS

### Literature search

**Figure 1:**
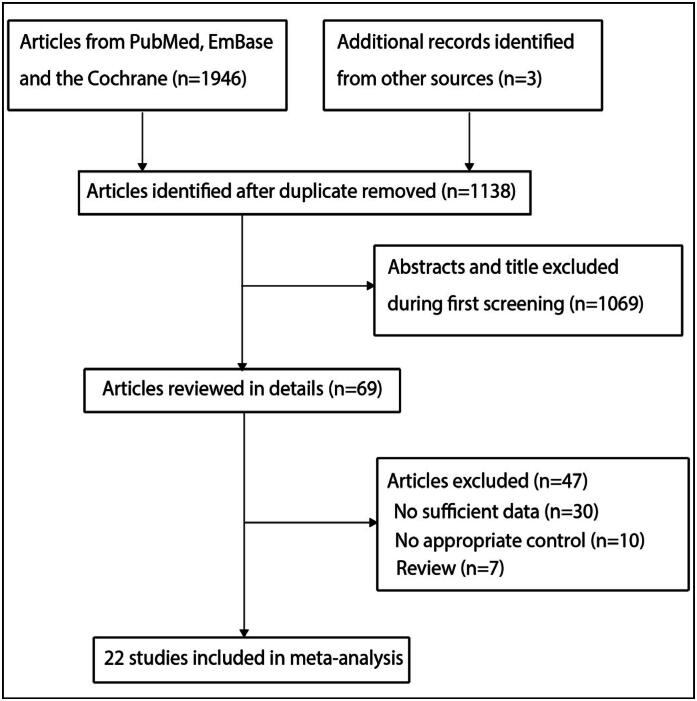
PRISMA flowchart of the processes of literature search and study selection.

A total of 1,946 articles were obtained from the electronic search, of which 1,138 were retained after removing duplicate articles. Additional 1,069 studies were excluded due to irrelevant topics. Further full-text review was performed on the remaining 69 studies, which resulted in the removal of 47 articles due to missing sufficient data (n = 30), lack of appropriate control subjects (n = 10), and review papers (n = 7). No new eligible trials were obtained from the review of the reference lists of relevant studies. Ultimately, 22 RCTs were included in the final meta-analysis (Figure 1).^20-41^

### Study characteristics

The characteristics of the selected trials and individuals are presented in Table 1. The number of individuals included was 70,837, and the sample size ranged from 50 to 36,282. Two trials were conducted in China and the remaining 21 in Western countries (the USA, Europe, and Australia). Of the trials, 15 included women and the remaining 8 included both men and women. Furthermore, 14 trials reported the effects of combined calcium and vitamin D supplementation, and the remaining 9 trials reported calcium supplementation alone. The follow-up duration ranged from 9 months to 7 years. The quality assessments in each trial are presented in Table 1.

**Table 1:** The characteristics of included trials and involved participants.

Study	Country	Sample size	Age (years)	Male (%)	Prior fracture	Vitamin D	Baseline 25 OHD	Intervention	Control	Follow-up	Jadad
Inkovaar^20^	Finland	130 (88/42)	79.8	17.9/18.7	NA	(1000 IU/d)	NA	Calcium (1.2 g/d)	Placebo	9.0 months	3
Hansson^21^	Sweden	50 (25/25)	65.9	0.0	Yes	No	NA	Calcium (1.0 g/d)	Placebo	3.0 years	2
Chapuy^22^	France	3,270 (1,634/1,636)	84.0	0.0	No	(800 IU/d)	NA	Calcium (1.2 g/d)	Placebo	1.5 years	4
Reid^23^	New Zealand	122 (61/61)	58.0	0.0	No	No	37.5	Calcium (1.0 g/d)	Placebo	4.0 years	4
Recker^24^	USA	191 (91/100)	73.5	0.0	Partial	No	25.5	Calcium (1.2 g/d)	Placebo	4.0 years	3
Dawson-Hughes^25^	USA	389 (187/202)	71.1	46.0	NA	(700 IU/d)	29.6	Calcium (0.5 g/d)	Placebo	3.0 years	4
Riggs^26^	USA	236 (119/117)	66.2	0.0	No	No	30.1	Calcium (1.6 g/d)	Placebo	4.0 years	2
Baron^27^	USA	930 (464/466)	61.0	72.3	NA	No	NA	Calcium (1.2 g/d)	Placebo	4.0 years	5
Peacock^28^	USA	253 (124/129)	73.8	28.4	Partial	No	25.0	Calcium (0.75 g/d)	Placebo	4.0 years	2
Chapuy^29^	France	583 (393/190)	85.2	0.0	No	(800 IU/d)	8.9	Calcium (1.2 g/d)	Placebo	2.0 years	4
Avenell^30^	UK	99 (64/35)	78.0	17.0	Yes	(800 IU/d)	NA	Calcium (1.0 g/d)	No treatment	3.8 years	3
Harwood^31^	UK	112 (75/37)	81.7	0.0	Yes	(800 IU/d)	11.9	Calcium (1.0 g/d)	No treatment	1.0 year	3
Porthouse^32^	UK	3,314 (1,321/1,993)	76.8	0.0	Partial	(800 IU/d)	NA	Calcium (1.0 g/d)	No treatment	2.1 years	3
Grant^33^	UK	3,949 (2,617/1,332)	77.3	15.3	Yes	(800 IU/d)	15.2	Calcium (1.0 g/d)	Placebo	2.0-5.2 years	5
Larsen^34^	Denmark	7,073 (4,957/2,116)	74.0	39.8	No	(400 IU/d)	NA	Calcium (1.0 g/d)	No treatment	3.0 years	3
Jackson^35^	USA	36,282 (18,176/18,106)	62.4	0.0	Partial	(400 IU/d)	18.9	Calcium (1.0 g/d)	Placebo	7.0 years	4
Prince^36^	Australia	1,460 (730/730)	75.2	0.0	Partial	No	31.0	Calcium (0.48 g/d)	Placebo	5.0 years	4
Reid^37^	New Zealand	1,471 (732/739)	74.3	0.0	Partial	No	20.7	Calcium (1.0 g/d)	Placebo	5.0 years	3
Bolton-Smith^38^	UK	123 (62/61)	68.6	0.0	NA	(400 IU/d)	23.9	Calcium (1.0 g/d)	Placebo	2.0 years	3
Salovaara^39^	Finland	3,195 (1,586/1,609)	67.3	0.0	Partial	(800 IU/d)	19.8	Calcium (1.0 g/d)	Placebo	3.0 years	3
Liu^40^	China	98 (50/48)	62.1	0.0	No	(600 IU/d)	NA	Calcium (1.5 g/d)	Placebo	1.0 year	2
Xue^41^	China	312 (139/173)	63.6	0.0	Partial	(800 IU/d)	30.8	Calcium (0.6 g/d)	Placebo	1.0 year	3

### Total fracture

**Figure 2:**
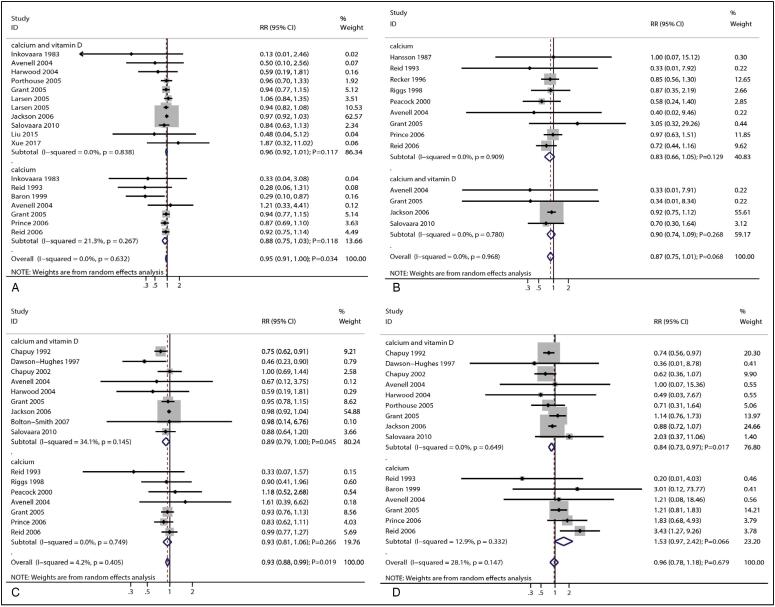
Effect of calcium supplementation on the risk of (A) total fracture, (B) vertebral fracture, (C) nonvertebral fracture, and (D) hip fracture.

**Figure 3:**
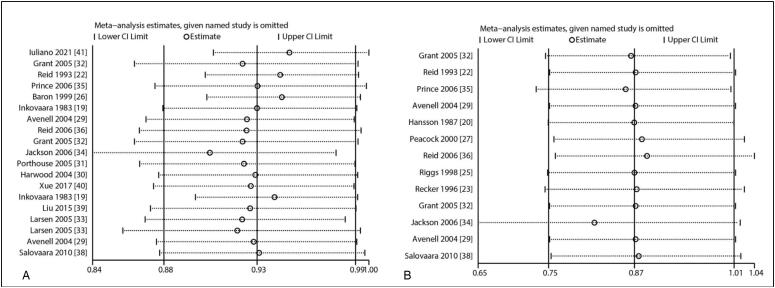
(A) Sensitivity analysis for the risk of total fracture. (B) Sensitivity analysis for the risk of vertebral fracture.

**Figure 4:**
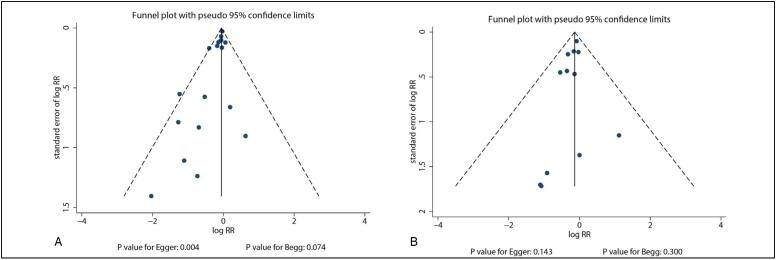
(A) Funnel plot for total fracture. (B) Funnel plot for vertebral fracture.

A total of 15 trials reported the effects of calcium supplementation on the risk of total fracture. The result indicated that the supplementation was associated with reduced risk of total fracture (RR: 0.93; 95% CI: 0.88–0.99; *P* = 0.019; Figure 2A), and there was no evidence of heterogeneity across the included trials (*I* ^2^ = 6.3%; *P* = 0.380). Sensitivity analysis revealed that the pooled conclusion was unaltered by the sequential removal of individual trials (Figure 3). On the other hand, subgroup analysis revealed that combined calcium supplementation was associated with reduced risk of total fracture when the mean age of individuals ≥ 70.0 years, history of fracture, irrespective of the calcium dose, did not combine with vitamin D, and irrespective of follow-up duration (Table 2). It was also observed that the reduced risk of total fracture in the subgroup of calcium dose ≥ 1.2 g was greater than that in the subgroup of calcium dose < 1.2 g (RR: 0.31; 95% CI: 0.13–0.73; *P* = 0.008). A potential publication bias was found for total fracture (*P*-value for Egger's test: 0.004; *P*-value for Begg's test: 0.074), and the pooled conclusion was unaltered when adjusted using the trim-and-fill method (Figure 4).

**Table 2:** Subgroup analyses for fracture at varies sites.

Outcomes	Factors	Subgroup	RR and 95%CI	P value	I2 (%)	P value for I2	RRR between subgroups	P value between subgroups
Total fracture	Mean age	≥ 70.0	0.92 (0.86-0.99)	0.031	0.0	0.662	1.11 (0.83-1.49)	0.494
		< 70.0	0.83 (0.62-1.10)	0.198	44.1	0.111		
	Sex	Female	0.96 (0.91-1.01)	0.081	0.0	0.673	1.07 (0.93-1.22)	0.359
		Both	0.90 (0.79-1.02)	0.112	30.6	0.164		
	Prior fracture	Yes	0.95 (0.90-0.99)	0.027	0.0	0.703	1.08 (0.81-1.44)	0.600
		No	0.88 (0.66-1.16)	0.357	51.1	0.085		
	Baseline 25 OHD	≥ 20.0	0.89 (0.76-1.04)	0.146	0.0	0.396	0.93 (0.79-1.09)	0.369
		< 20.0	0.96 (0.91-1.01)	0.127	0.0	0.785		
	Dose of calcium	≥ 1.2	0.29 (0.12-0.69)	0.005	0.0	0.918	0.31 (0.13-0.73)	0.008
		< 1.2	0.95 (0.91-0.99)	0.022	0.0	0.656		
	Combined with vitamin D	Yes	0.96 (0.92-1.01)	0.117	0.0	0.820	1.16 (0.98-1.37)	0.089
		No	0.83 (0.71-0.98)	0.030	31.9	0.173		
	Follow-up duration	≥ 3.0	0.95 (0.91-1.00)	0.041	0.0	0.491	1.20 (0.96-1.51)	0.110
		< 3.0	0.79 (0.63-0.98)	0.034	0.0	0.426		
	Study quality	High	0.88 (0.78-1.00)	0.058	51.6	0.053	0.94 (0.80-1.10)	0.410
		Low	0.94 (0.85-1.03)	0.171	0.0	0.820		
Vertebral fracture	Mean age	≥ 70.0	0.83 (0.65-1.05)	0.121	0.0	0.833	0.92 (0.68-1.25)	0.602
		< 70.0	0.90 (0.75-1.09)	0.291	0.0	0.946		
	Sex	Female	0.88 (0.76-1.03)	0.112	0.0	0.975	1.35 (0.62-2.94)	0.443
		Both	0.65 (0.30-1.37)	0.254	0.0	0.688		
	Prior fracture	Yes	0.87 (0.75-1.02)	0.083	0.0	0.935	1.07 (0.4-2.62)	0.875
		No	0.81 (0.34-1.97)	0.646	0.0	0.583		
	Baseline 25 OHD	≥ 20.0	0.82 (0.65-1.04)	0.109	0.0	0.881	0.90 (0.66-1.22)	0.505
		< 20.0	0.91 (0.75-1.11)	0.352	0.0	0.613		
	Dose of calcium	≥ 1.2	0.85 (0.58-1.25)	0.417	0.0	0.964	0.97 (0.64-1.47)	0.871
		< 1.2	0.88 (0.74-1.03)	0.111	0.0	0.918		
	Combined with vitamin D	Yes	0.90 (0.74-1.09)	0.292	0.0	0.789	1.08 (0.80-1.47)	0.600
		No	0.83 (0.66-1.05)	0.127	0.0	0.916		
	Follow-up duration	≥ 3.0	0.87 (0.75-1.01)	0.074	0.0	0.970	-	-
		< 3.0	-	-	-	-		
	Study quality	High	0.93 (0.78-1.11)	0.433	0.0	0.768	1.22 (0.89-1.69)	0.217
		Low	0.76 (0.58-0.99)	0.043	0.0	0.990		
Non-vertebral fracture	Mean age	≥ 70.0	0.88 (0.80-0.97)	0.009	2.7	0.417	0.91 (0.81-1.01)	0.087
		< 70.0	0.97 (0.92-1.03)	0.391	0.0	0.674		
	Sex	Female	0.91 (0.84-1.00)	0.046	14.5	0.309	0.99 (0.84-1.17)	0.897
		Both	0.92 (0.80-1.06)	0.253	3.2	0.396		
	Prior fracture	Yes	0.97 (0.92-1.02)	0.203	0.0	0.944	1.21 (1.01-1.46)	0.044
		No	0.80 (0.67-0.96)	0.018	5.4	0.366		
	Baseline 25 OHD	≥ 20.0	0.87 (0.71-1.05)	0.147	7.9	0.368	0.90 (0.73-1.10)	0.292
		< 20.0	0.97 (0.92-1.02)	0.271	0.0	0.917		
	Dose of calcium	≥ 1.2	0.80 (0.68-0.95)	0.009	0.0	0.380	0.83 (0.70-0.99)	0.041
		< 1.2	0.96 (0.91-1.01)	0.137	0.0	0.636		
	Combined with vitamin D	Yes	0.89 (0.79-1.00)	0.052	35.0	0.138	0.96 (0.80-1.14)	0.630
		No	0.93 (0.81-1.06)	0.260	0.0	0.752		
	Follow-up duration	≥ 3.0	0.96 (0.91-1.01)	0.144	0.0	0.614	1.22 (1.02-1.45)	0.031
		< 3.0	0.79 (0.67-0.94)	0.007	0.0	0.540		
	Study quality	High	0.89 (0.79-0.99)	0.040	49.4	0.054	0.94 (0.76-1.16)	0.546
		Low	0.95 (0.79-1.13)	0.551	0.0	0.960		
Hip fracture	Mean age	≥ 70.0	0.92 (0.69-1.22)	0.559	49.7	0.025	1.03 (0.73-1.46)	0.851
		< 70.0	0.89 (0.73-1.08)	0.236	0.0	0.490		
	Sex	Female	0.90 (0.68-1.20)	0.481	44.8	0.070	0.98 (0.61-1.57)	0.928
		Both	0.92 (0.63-1.35)	0.682	36.8	0.148		
	Prior fracture	Yes	1.02 (0.77-1.35)	0.886	46.8	0.043	1.44 (0.99-2.08)	0.056
		No	0.71 (0.56-0.91)	0.007	0.0	0.618		
	Baseline 25 OHD	≥ 20.0	1.76 (0.69-4.48)	0.237	31.9	0.221	1.85 (0.71-4.82)	0.206
		< 20.0	0.95 (0.78-1.16)	0.612	16.4	0.308		
	Dose of calcium	≥ 1.2	0.72 (0.56-0.92)	0.008	0.0	0.580	0.72 (0.50-1.04)	0.081
		< 1.2	1.00 (0.76-1.31)	0.984	40.4	0.065		
	Combined with vitamin D	Yes	0.84 (0.73-0.97)	0.018	0.0	0.655	0.69 (0.36-1.33)	0.270
		No	1.21 (0.64-2.27)	0.554	65.8	0.007		
	Follow-up duration	≥ 3.0	1.14 (0.89-1.47)	0.306	21.4	0.239	1.70 (1.23-2.36)	0.001
		< 3.0	0.67 (0.54-0.82)	< 0.001	0.0	0.808		
	Study quality	High	0.85 (0.69-1.05)	0.139	42.0	0.078	0.62 (0.30-1.30)	0.204
		Low	1.37 (0.68-2.79)	0.381	22.6	0.264		

### Vertebral fracture

A total of 11 trials reported the effects of calcium supplementation on the risk of vertebral fracture. It was found that the supplementation did not exert a significant effect on the risk of vertebral fracture (RR: 0.87; 95% CI: 0.75–1.01; *P* = 0.074; Figure 2B), and there was no evidence of heterogeneity among the included trials (*I* ^2^ = 0.0%; *P* = 0.970). The pooled conclusion was solid and unaltered by the sequential removal of individual studies (Figure 3). Subgroup analysis revealed that calcium supplementation was associated with reduced risk of vertebral fracture in pooled trials with moderate quality (Table 2). In addition, no significant publication bias was observed for vertebral fracture (*P*-value for Egger's test: 0.143; *P*-value for Begg's test: 0.300; Figure 4).

### Nonvertebral fracture

A total of 14 trials reported the effects of calcium supplementation on the risk of nonvertebral fracture. It was observed that the supplementation significantly reduced the risk of nonvertebral fracture (RR: 0.93; 95% CI: 0.87–0.99; *P* = 0.023; Figure 2C). No significant heterogeneity was found for nonvertebral fracture across the included trials (*I* ^2^ = 5.3%; *P* = 0.393). The pooled conclusion was variable due to the marginal 95% CI (Figure 3). Subgroup analysis revealed that calcium supplementation was associated with reduced risk of nonvertebral fracture for individuals with a mean age of ≥70.0 years, female sex, no history of fracture, calcium dose ≥ 1.2 g, follow-up duration < 3.0 years, and studies with high quality (Table 2). Furthermore, the effects of calcium supplementation on the risk of nonvertebral fracture could be affected by a history of fracture (RR: 1.21; 95% CI: 1.01–1.46; *P* = 0.044), calcium dose (RR: 0.83; 95% CI: 0.70–0.99; *P* = 0.041), and follow-up duration (RR: 1.22; 95% CI: 1.02–1.45; *P* = 0.031). No significant publication bias for nonvertebral fracture was observed (*P*-value for Egger's test: 0.081; *P*-value for Begg's test: 0.964; Figure 4).

### Hip fracture

A total of 14 trials reported the effects of calcium supplementation on the risk of hip fracture. The result indicated that the supplementation was not associated with the risk of hip fracture (RR: 0.90; 95% CI: 0.73–1.12; *P* = 0.355; Figure 2D), and significant heterogeneity was observed across the included trials (*I* ^2^ = 38.4%; *P* = 0.059). Sensitivity analysis revealed that the pooled conclusion was unaltered by the exclusion of any particular trial (Figure 3). Subgroup analysis revealed that calcium could protect against the risk of hip fracture for patients with no history of fracture, calcium dose ≥ 1.2 g, vitamin D supplementation, and follow-up duration < 3.0 years (Table 2). No significant publication bias was observed for hip fracture (*P*-value for Egger's test: 0.521; *P*-value for Begg's test: 0.893; Figure 4).

## DISCUSSION

In this study, 23 RCTs were analyzed to determine the effect of calcium supplementation on the risk of total, vertebral, nonvertebral, and hip fractures. The results indicated that calcium supplementation can significantly reduce the risk of total and nonvertebral fractures whereas it does not have an effect on the risk of vertebral and hip fractures. The effect of calcium supplementation on the risk of total fracture could be affected by the calcium dose whereas that on the risk of nonvertebral fracture could be affected by a history of fracture, calcium dose, and follow-up duration. On the other hand, the effect of calcium supplementation on the risk of hip fracture could be affected by follow-up duration.

Several systematic reviews and meta-analyses have demonstrated the effect of calcium supplementation on the risk of fracture. Weaver et al. conducted a meta-analysis of eight studies and reported that combined calcium and vitamin D supplementation was associated with reduced risk of total and hip fractures. They suggested that calcium and vitamin D supplementation be given to both community-dwelling and institutionalized middle-aged to older adults.^42^ However, they did not investigate the effects of calcium supplementation alone and those of combined calcium and vitamin D supplementation on the risk of fracture in a specific subpopulation. Zhao et al. conducted a meta-analysis of 33 RCTs to determine the effect of calcium or vitamin D supplementation on the risk of fracture in community-dwelling older adults. They reported that calcium, vitamin D, or the combined use of both did not affect the risk of fracture among these individuals.^43^ This nonsignificant effect of calcium supplementation alone or the combined calcium and vitamin D supplementation might vary according to the pooled analysis. Therefore, the present meta-analysis was conducted to determine the effect of calcium supplementation on the risk of fracture in older adults.

As reported in previous meta-analyses, calcium supplementation could exert a protective effect against the risk of total fracture. Majority of the included trials did not observe a significant difference between calcium and placebo in the effect on the risk of total fracture, whereas a trial conducted by Baron et al. found that calcium supplementation was associated with reduced risk of total fracture.^27^ This trial used recurrent colorectal adenomas as the primary endpoint, and this significant effect may have occurred incidentally. Subgroup analysis revealed that calcium supplementation could exert a protective affect against the risk of total fracture in individuals with a mean age of ≥70.0 years, history of fracture, irrespective of calcium dose, did not combine with vitamin D, and irrespective of follow-up duration. Consistent with a previous meta-analysis,^42^ this study found that calcium supplementation had no significant effect on the risk of vertebral fractures, regardless of the use or nonuse of vitamin D. All the trials pooled for this study had similar conclusions; in addition, the number of events that occurred was lower than expected, and broad 95% CI values were obtained.

The results of this study suggest that calcium supplementation significantly reduces the risk of nonvertebral fractures, particularly when used in combination with vitamin D. This is probably because vitamin D promotes calcium absorption in the gut whereas serum calcium maintains normal bone mineralization.^42^ Furthermore, the benefits of calcium supplementation were mainly detected in the subgroups of individuals with a mean age of ≥70.0 years, female sex, no history of fracture, calcium dose ≥ 1.2 g, follow-up duration < 3.0 years, and studies with high quality. These results suggested that the beneficial effects of calcium supplementation on the risk of nonvertebral fracture were more evident in individuals with the aforementioned characteristics. Contrary to the previous meta-analysis, the present study did not find evidence to support that calcium supplementation can reduce the risk of hip fractures. This discrepancy could be attributed mainly to the fact that the studies included in the previous meta-analysis used combined calcium and vitamin D supplementation.^42^ Subgroup analysis demonstrated the beneficial effect of calcium supplementation on the risk of hip fracture mainly in individuals with no history of fracture, calcium dose ≥ 1.2 g, combined use of vitamin D, and follow-up duration < 3.0 years. The reason for this could be that these characteristics were associated with a greater requirement for calcium, which could slow the progression of hip fracture.

This study has the following limitations that need to be acknowledged: (1) the inclusion of different population characteristics can lead to variations in the intake of calcium or vitamin D through food, which can influence the occurrence of fractures; (2) most trials did not define fracture as a primary endpoint, and the power was not sufficient to detect the difference between calcium and placebo; and (3) there were inherent limitations for meta-analysis based on published articles, including inevitable publication bias and restricted detailed analyses.

In conclusion, this study demonstrates that calcium supplementation exerts a protective effect against the risk of total and nonvertebral fractures. However, the long-term effects of calcium on the risk of fracture require further investigation, and the dose–response for background vitamin D use should be identified in older adults.
